# Lipase hypersecretion syndrome: A rare cutaneous manifestation of advanced pancreatic acinar cell carcinoma

**DOI:** 10.1002/ccr3.2785

**Published:** 2020-03-12

**Authors:** Wasay Nizam, Adil A. Shah, Fareed Rajack, Asa Ramdath, Tammey Naab, Mallory Williams

**Affiliations:** ^1^ Department of Surgery Howard University College of Medicine Washington DC USA; ^2^ Department of Pathology Howard University College of Medicine Washington DC USA

**Keywords:** acinar cell carcinoma, lipase hypersecretion syndrome, neoplastic syndrome, pancreatic panniculitis

## Abstract

Careful recognition of cutaneous lesions in patients with malignancies may aid in avoiding additional morbidity during end of life care.

## Graphical abstract

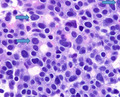

## 1 INTRODUCTION

Pancreatic panniculitis is a rare cutaneous manifestation of a variety of pancreatic diseases.^1^ When seen in conjunction with polyarthritis and eosinophilia, it is termed lipase hypersecretion syndrome (LHS), a paraneoplastic syndrome most commonly seen in the setting of pancreatic acinar cell carcinoma.^2^ LHS is a neoplastic disorder resulting from exocrine excess, with the release of enzymes into circulation, a phenomenon that has been described as “endocrine‐ization of an exocrine function.”^2^ First reported by Berner in 1908 who described a “functional acinar cell” malignancy accompanied by this constellation of findings^3^; the occurrence of these findings has been attributed to the enzymatic properties on distant tissues of high circulating levels of lipase within peripheral circulation.^4^ A recent review article found that since this initial report, further observations of this pathology have been limited to a few case reports.^2^ Given this rarity and infrequent presentation, LHS remains a poorly studied subject that may often be initially misdiagnosed or unrecognized^2^ This is important to consider as peripheral signs of pancreatic malignancies may often precede the development of locoregional effects by weeks,^5^ thus providing an opportunity for early diagnosis. Our ensuing review will include a case report, followed by an overview of the syndromes’ clinical manifestations, pathophysiology, histological features, and management principles pertinent to surgical care.

## 2 CASE HISTORY/EXAMINATION

**Figure 1:**
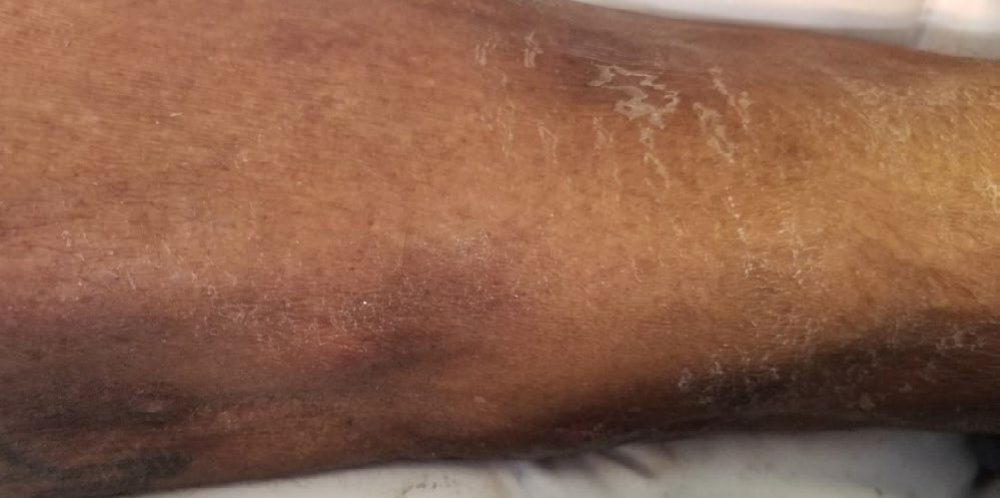
Characteristic extremity nodules of LHS

**Figure 2:**
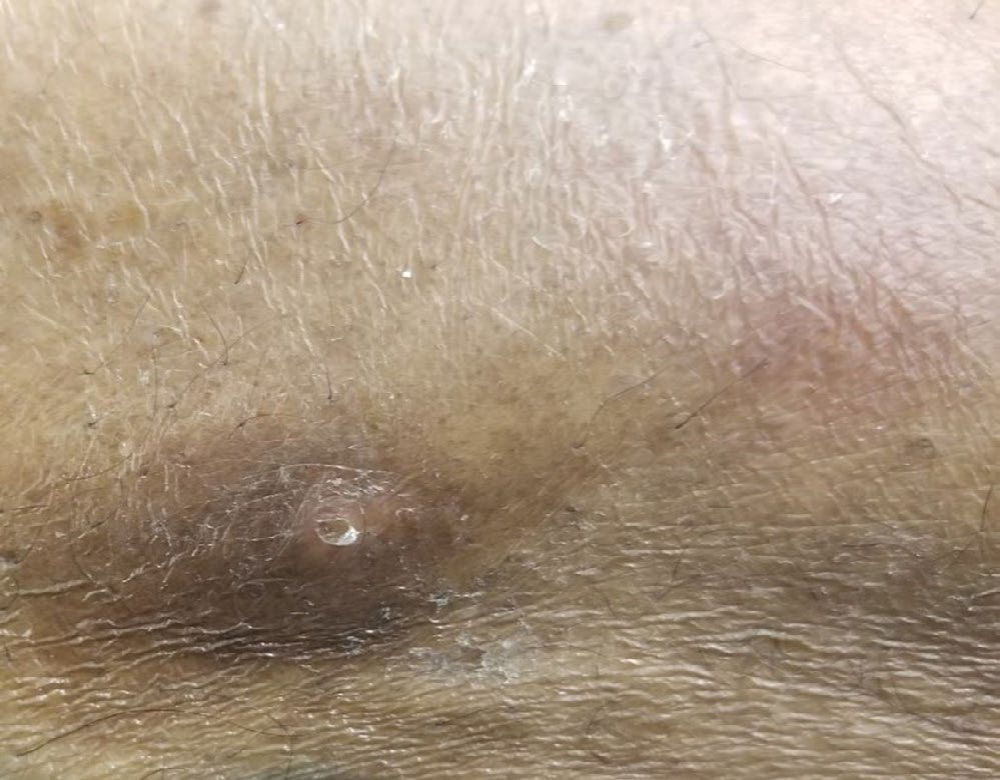
Characteristic extremity nodules of LHS

Our patient was a 69‐year‐old man who presented to the hospital with multiple painful nodular lesions on his legs bilaterally. These lesions were first noticed one month prior to presentation on his right lower limb but had progressed over time to now involve both legs and were associated with worsening pain and serous discharge. He was not associated with fevers or chills; however, he did admit to loss of appetite, weight loss, and malaise. Past medical history was significant for hypertension, Hepatitis C, and prostate cancer managed with brachytherapy. Physical examination revealed a 3 × 3 cm tender nodule on anteromedial aspect of right leg draining seropurulent fluid as well as several 1‐2 cm nodules on bilateral legs, hyperpigmented and tender to palpation. (Figures 1 and 2).

## 3 DIFFERENTIAL DIAGNOSIS, INVESTIGATIONS, AND TREATMENT

**Figure 3:**
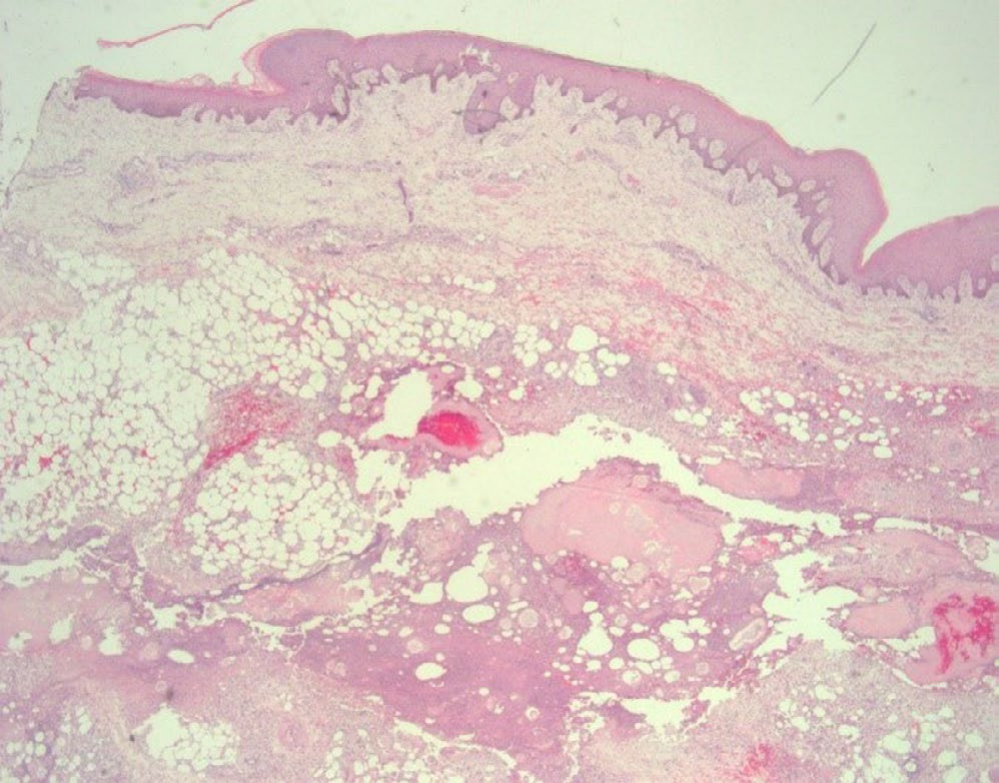
40× magnification of fat necrosis surrounded by neutrophils and foamy histiocytes consistent with pancreatic panniculitis

An excisional biopsy of the prominent right leg nodular lesion was performed, and histopathologic findings included subcutaneous enzymatic fat necrosis surrounded by neutrophils and foamy histiocytes and epidermal necrosis consistent with pancreatic panniculitis (Figure 3). This prompted a workup for pancreatic pathology including blood and radiological investigations, upper and lower endoscopies.

Pertinent laboratories included serum lipase (5444 IU/L), carbohydrate antigen 19‐9 (CA 19‐9) (44 U/mL), carcinoembryonic antigen (CEA) (0.5 ng/mL).

**Figure 4:**
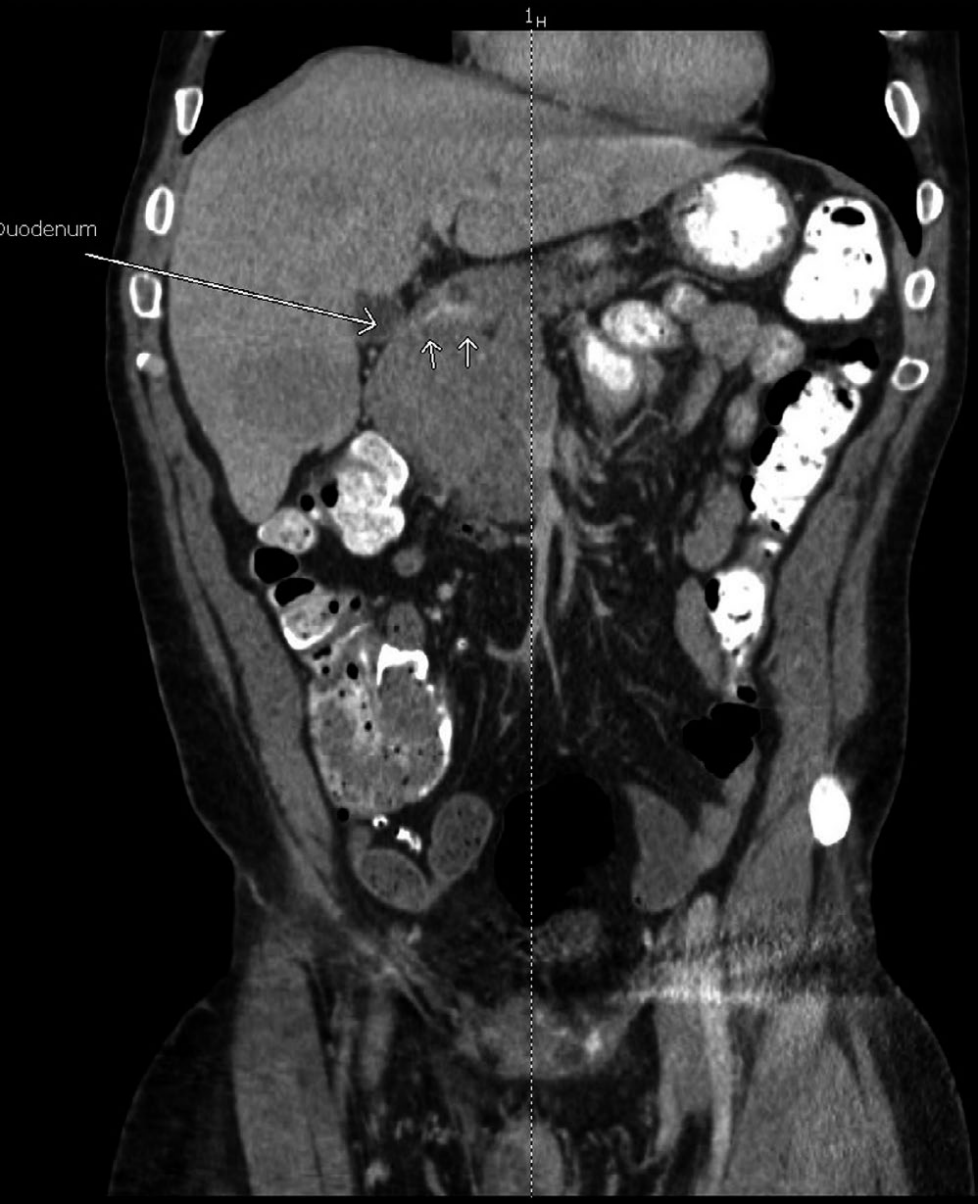
Coronal and axial cuts of an abdominal CT demonstrating pancreatic mass with duodenal invasion

**Figure 5:**
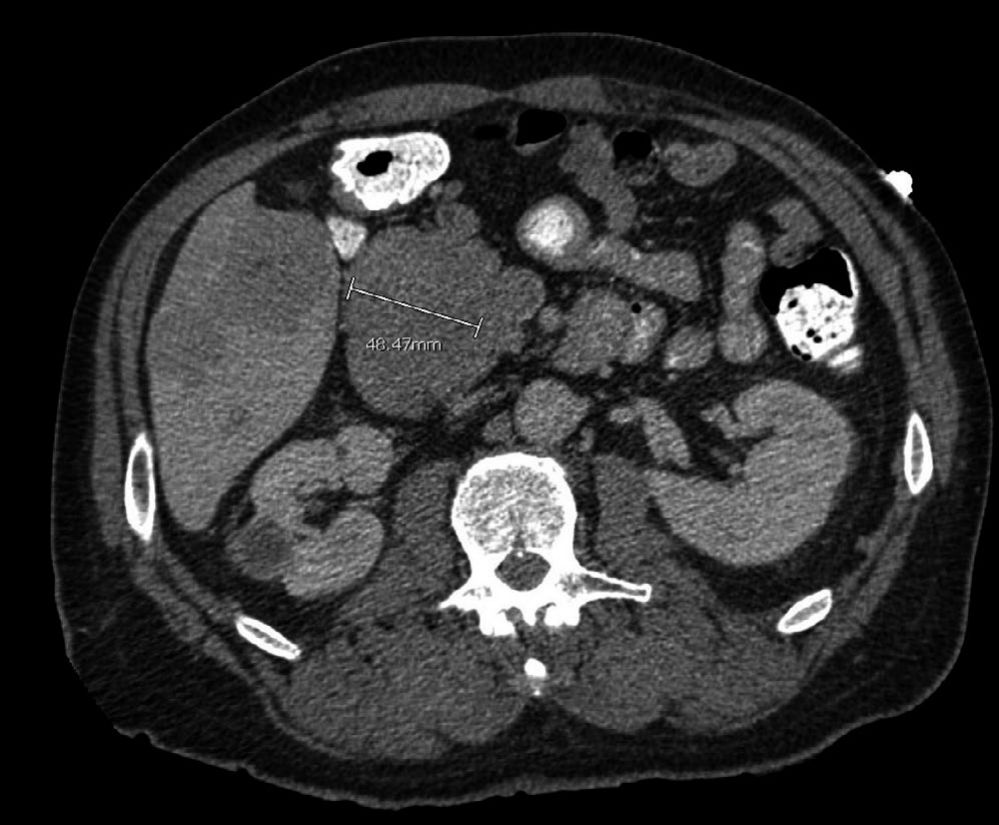
Coronal and axial cuts of an abdominal CT demonstrating pancreatic mass with duodenal invasion

Computed tomography of the abdomen revealed an 8.7 × 6.2 × 5.7 cm mass arising from the pancreatic head (Figures 4 and 5) causing significant mass effect upon the second portion of the duodenum. Decreased attenuation of the portal vein and the superior mesenteric vein was suggestive of invasion. There was no pancreatic duct, common bile duct, or intrahepatic biliary ductal dilatation. Multiple large hepatic lesions showing peripheral enhancement were noted, with the largest lesion measuring 5.1 cm in greatest dimension consistent with metastatic disease. These suspicious masses in the liver were biopsied percutaneously by interventional radiology in order to obtain a definitive tissue diagnosis and to aid in formulating a therapeutic plan.

**Figure 6:**
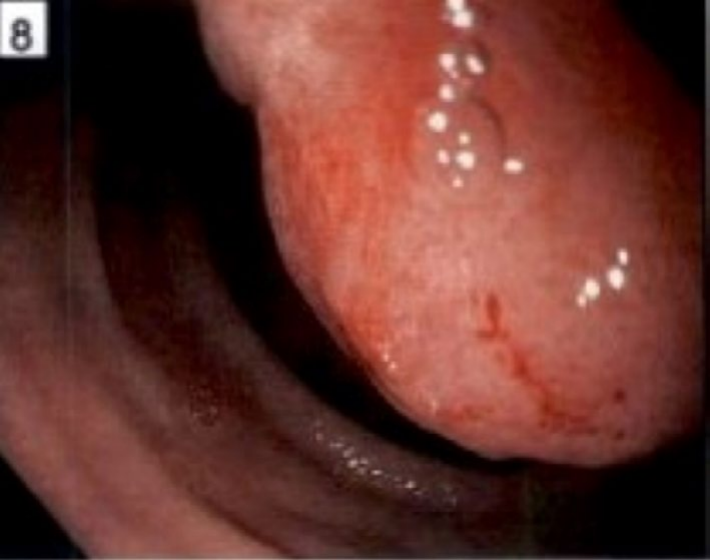
Endoscopic image of pancreatic mass with duodenal invasion

An upper endoscopy was performed, and a large (approximately 5 cm) duodenal mass with ulceration extending from the bulb toward the second portion of the duodenum was seen and biopsied (Figure 6).

**Figure 7:**
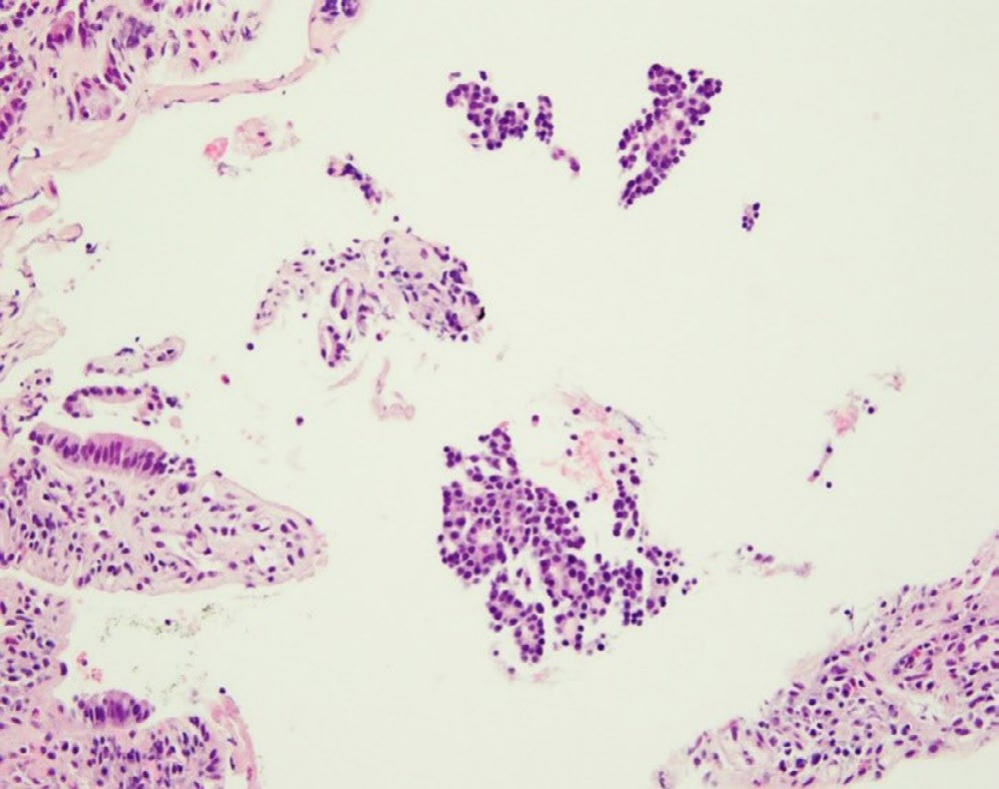
100× magnification of duodenal biopsy with collections of carcinoma with uniform cells and tubular formations

**Figure 8:**
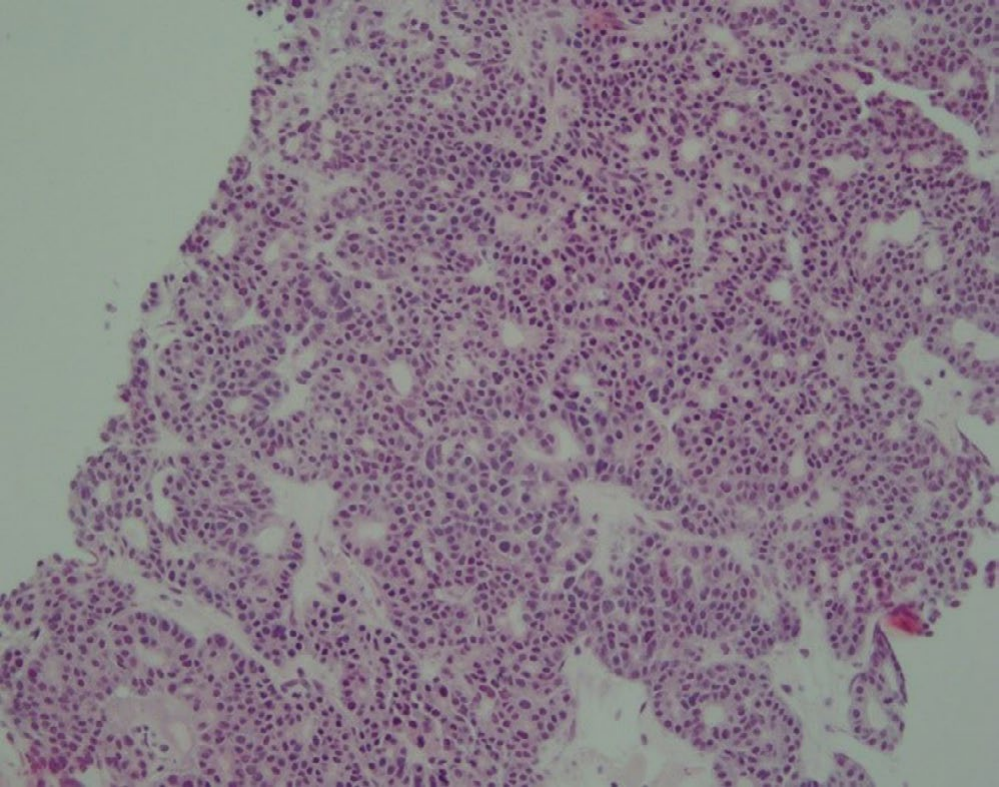
100× magnification of metastatic carcinoma in liver composed of high‐grade carcinoma forming sheets and tubular formations

**Figure 9:**
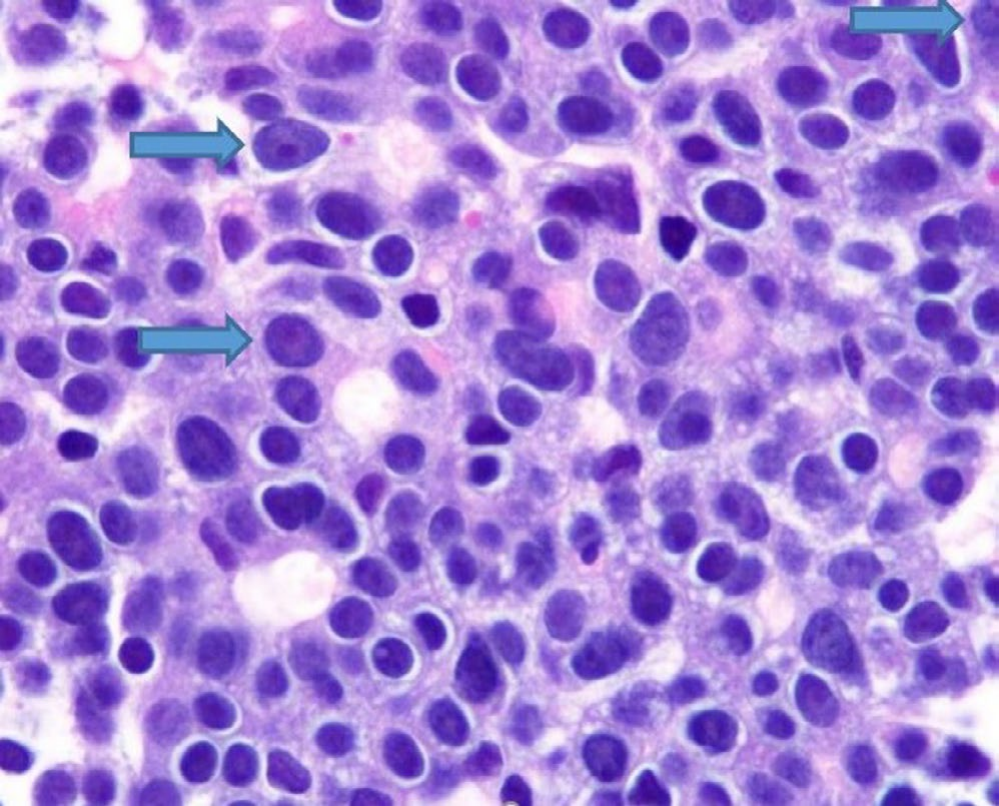
Liver biopsy (1000× magnification) showing fine chromatin, large cherry red nucleoli (arrows) and nuclear pleomorphism

Duodenal and liver biopsy results were concordant with the diagnosis of pancreatic acinar cell carcinoma (see Figures 7, 8, 9). Factors that supported this diagnosis included the strong diffuse cytoplasmic expression of alpha1‐antichymotrypsin, alpha‐1 antitrypsin and chymotrypsin and negative CD‐56 and chromogranin. Synaptophysin immunostaining showed patchy weak expression. Glypican‐3 was negative. Periodic acid‐Schiff (PAS) stain with diastase was noncontributory. After a thorough multidisciplinary team workup including pathology, a diagnosis of lipase hypersecretion syndrome secondary to Stage 4 pancreatic acinar cell carcinoma was made.

**Figure 10:**
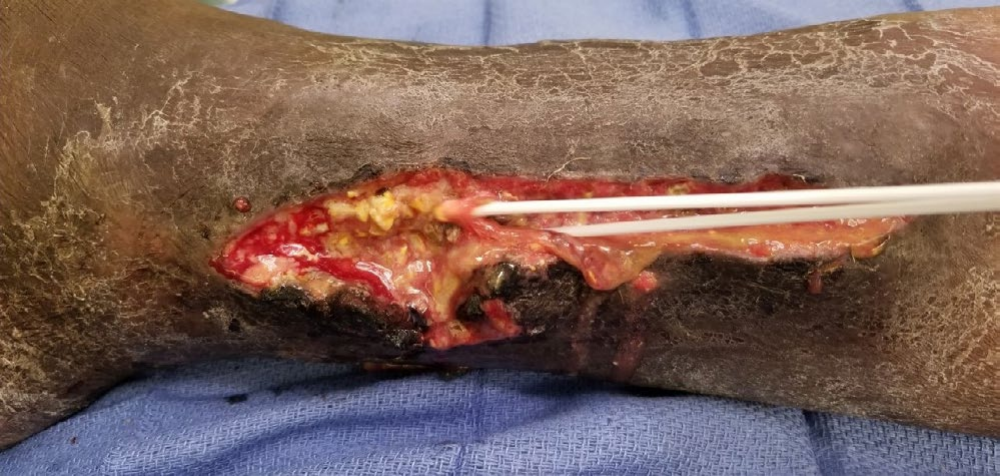
Gross picture of pancreatic panniculitis with characteristic discharge

A large portion of his care centered on the management of his chronic nonhealing lower extremity wounds. After serial debridements, washouts, and extensive wound care, he developed multiple lower extremity wounds with the largest measuring 8 × 15 cm in size. These were managed with a combination of routine dressing changes, application of negative pressure therapy, and use of a‐cellular matrix. These wounds were extremely slow to heal due to the patient's overall physiologic condition and did respond to aggressive wound care (Figure 10).

## 4 OUTCOME AND FOLLOW‐UP

After a multidisciplinary discussion at tumor board within the Howard University Cancer Center, the patient was offered a management plan centering on palliative care. His poor performance status, however, prohibited the use of chemotherapy. The patient required serial transfusions for chronic gastrointestinal hemorrhage secondary to the pancreatic tumor invasion into the duodenum, and palliative radiotherapy was offered however the family declined. After consideration of his treatment options, the patient and his family had expressed their wish to pursue hospice care with continued palliative treatments largely due to the extremely poor prognosis of his neoplastic condition. He continued to receive appropriate inpatient nutritional support, wound care and management of his comorbid medical conditions with the goal of transitioning his care to a home‐based palliative care system. However, prior to discharge, the patient succumbed to the nature of his end‐stage disease. This represented a total of 2 months overall survival from the time of his diagnosis.

## 5 DISCUSSION

Acinar cell carcinoma (ACC) is an extremely rare pancreatic tumor comprising 1%‐2% of pancreatic tumors^6^ with 50% of tumors presenting with metastases at the time of diagnosis and those patients with advanced disease (Stage III and IV) having very poor prognosis.^6^, ^7^ Cross‐sectional imaging modalities such as computed tomography and magnetic resonance imaging that are enhanced with contrast agents are often utilized to define the extent of primary lesions and possible metastatic disease.^8^, ^9^ An estimated 10%‐15% of ACC’s manifest the neoplastic exocrine disorder termed LHS,^10^ and typically, this occurs at an advanced stage with most patients having concurrent metastatic disease to the liver.^2^ Other malignancies that may manifest in this manner include pancreatic islet cell^11^ and neuroendocrine tumors^12^ as well as adrenal neuroendocrine carcinoma.^4^ The abnormally high levels of lipase present in this condition may affect a variety of organs with varying degrees of uncontrolled lipolysis.^13^ This is most notable in subcutaneous tissues where enzymatic damage to subcutaneous adipocytes results in fat necrosis and a subsequent inflammatory process.^1^ Resolution of the inflammatory phase is followed by regression of these lesions or formation of fibrocystic nodules.^2^ Subsequent enlargement of these areas may produce compressive effects manifesting as erythematous or purplish‐brown epithelial discoloration, erosion, and ulceration^13^ that may have an oily brown discharge.^14^ Commonly, the lower extremities are afflicted, but lesions have also been described on the trunk and scalp as well.^15^ A biopsy may be obtained for confirmation with pathognomonic findings described as anucleate “ghost” cells consisting of granular debris with an eosinophilic rim; these “ghost” cells may be calcified and are often surrounded by neutrophils and fat necrosis with accompanying hemorrhage.^16^

Additionally, lipolysis of periarticular adipose tissue in joints and intramedullary adipose tissue induces intra‐articular inflammation. This results in painful swollen joints,^13^ typically the knees and ankles^17^ and in a symmetrical manner.^14^ This inflammatory tissue may also act as a nidus for infection with cultures typically yielding staphylococcal aureus or epidermis.^13^ Cross‐sectional imaging of involved joints may demonstrate loss of joint spaces, osteolytic lesions, and periostitis.^18^

As many of these patients present with late‐stage disease, thereby precluding them from surgical resection, much of their care is directed toward symptom relief and palliation. Successful chemotherapy regimens mentioned have used FOLFIRINOX.^19^ Successful elimination of liver metastases with chemoembolization using a regimen of mitomycin, DSM450, cisplatin, and Lipiodol has also been noted to have drastic improvement in LHS symptoms.^20^ For infected lesions, following drainage, tetracyclines may be a potential antibiotic choice as they have been shown to decrease lipase secretion^21^ with resolution of subcutaneous lesions in one case.^22^ As in our patient, there may be a resulting huge wound burden for which care can be a source of significant pain, discomfort, and metabolic demand in patients who are already burdened by malnourishment and supraphysiologic demands. In this context, it is important for caregivers to recognize the nature, character, and consistency of discharge from foci of pancreatic panniculitis which may frequently be misdiagnosed as infectious or inflammatory nodules (Figures 1, 2, and 10). Appropriate recognition of these lesions is crucial as this can guide diagnostic approach toward punch biopsies as opposed to excisional biopsies, thus avoiding additional morbidity during end of life care. We utilized several approaches for optimizing wound care with the use of negative pressure wound therapy and noncrosslinked wound scaffold matrices to augment healing. In the overall clinical setting, these results were highly promising and serve as a potential guide for similar cases.

In conclusion, lipase hypersecretion syndrome is a rare entity associated with various pancreatic diseases. It should be considered in suspicious lesions that present diagnostic challenges. Early recognition can help initiation of appropriate multidisciplinary care for adequate symptomatic relief and reduced morbidity.

## CONFLICT OF INTEREST

The authors declare that they have no competing interests and there are no relevant financial disclosures to report.

## AUTHOR CONTRIBUTION

Wasay Nizam: made substantial contributions to conception, design, data acquisition and interpretation of the study and involved in initial drafting of the manuscript and critically revised the final manuscript. Adil Aijaz Shah, Fareed Rajack, and Asa Ramdath: made substantial contributions to conception and design of the study and involved in initial drafting of the manuscript and critically revised the final manuscript. Tammey Naab: made substantial contributions to conception and design of the study and involved in initial drafting of the manuscript and critically revised the final manuscript for important intellectual content. Mallory Williams: made substantial contributions to conception and design of the study, acquisition, analysis, and interpretation of data and involved in initial drafting of the manuscript and critically revised the final manuscript for important intellectual content. All authors: reviewed the final manuscript and give approval of the version to be published and agree to be accountable for all aspects of the work in ensuring that questions related to the accuracy or integrity of any part of the work are appropriately investigated and resolved, and critically reviewed and provided feedback to the entire manuscript. All authors: read and approved the final manuscript.

## CONSENT

Written informed consent was obtained from the patient's family prior to submission.
